# Effects of Venlafaxine, Risperidone and Febuxostat on Cuprizone-Induced Demyelination, Behavioral Deficits and Oxidative Stress

**DOI:** 10.3390/ijms22137183

**Published:** 2021-07-02

**Authors:** Dragos Paul Mihai, Anca Ungurianu, Cosmin I. Ciotu, Michael J. M. Fischer, Octavian Tudorel Olaru, George Mihai Nitulescu, Corina Andrei, Cristina Elena Zbarcea, Anca Zanfirescu, Oana Cristina Seremet, Cornel Chirita, Simona Negres

**Affiliations:** 1 Faculty of Pharmacy, “Carol Davila”, University of Medicine and Pharmacy, 020956 Bucharest, Romania; dragos_mihai@drd.umfcd.ro (D.P.M.); octavian.olaru@umfcd.ro (O.T.O.); george.nitulescu@umfcd.ro (G.M.N.); corina.andrei@umfcd.ro (C.A.); cristina.zbarcea@umfcd.ro (C.E.Z.); anca.zanfirescu@umfcd.ro (A.Z.); oana.seremet@umfcd.ro (O.C.S.); cornel.chirita@umfcd.ro (C.C.); simona.negres@umfcd.ro (S.N.); 2 Center for Physiology and Pharmacology, Institute of Physiology, Medical University of Vienna, 1090 Vienna, Austria; ionut.ciotu@meduniwien.ac.at (C.I.C.); michael.jm.fischer@meduniwien.ac.at (M.J.M.F.)

**Keywords:** multiple sclerosis (MS) model, neurodegenerative diseases, desvenlafaxine, paliperidone, TRPA1 ligand, oxidative stress, motor performance, cold sensitivity

## Abstract

Multiple sclerosis (MS) is a demyelinating, autoimmune disease that affects a large number of young adults. Novel therapies for MS are needed considering the efficiency and safety limitations of current treatments. In our study, we investigated the effects of venlafaxine (antidepressant, serotonin-norepinephrine reuptake inhibitor), risperidone (atypical antipsychotic) and febuxostat (gout medication, xanthine oxidase inhibitor) in the cuprizone mouse model of acute demyelination, hypothesizing an antagonistic effect on TRPA1 calcium channels. Cuprizone and drugs were administered to C57BL6/J mice for five weeks and locomotor activity, motor performance and cold sensitivity were assessed. Mice brains were harvested for histological staining and assessment of oxidative stress markers. Febuxostat and metabolites of venlafaxine (desvenlafaxine) and risperidone (paliperidone) were tested for TRPA1 antagonistic activity. Following treatment, venlafaxine and risperidone significantly improved motor performance and sensitivity to a cold stimulus. All administered drugs ameliorated the cuprizone-induced deficit of superoxide dismutase activity. Desvenlafaxine and paliperidone showed no activity on TRPA1, while febuxostat exhibited agonistic activity at high concentrations. Our findings indicated that all three drugs offered some protection against the effects of cuprizone-induced demyelination. The agonistic activity of febuxostat can be of potential use for discovering novel TRPA1 ligands.

## 1. Introduction

Multiple sclerosis (MS) is a chronic, debilitating autoimmune disorder of the central nervous system (CNS) which occurs in a significant number of young adults worldwide, affecting 30 per 100,000 persons according to WHO [1,2]. The underlying pathological mechanisms responsible for CNS lesions are mediated by reactive lymphocytes that cross the blood-brain barrier (BBB) and are thereafter activated. The adaptive immune response generates chronic neuroinflammation, which leads to demyelination, loss of mature oligodendrocytes, axonal injury, oxidative stress, excitotoxicity, astrogliosis and microgliosis [3,4,5]. Current therapeutic strategies for MS include immunomodulatory molecules (i.e., glatiramer acetate, dimethyl fumarate, fingolimod, mitoxantrone) and monoclonal antibodies for relapsing-remitting forms (natalizumab, ocrelizumab, alemtuzumab) [6,7,8,9,10,11]. These approved small molecules and biologic therapies have limited efficacy, high costs and an unfavorable safety profile, hence the need for discovering novel therapeutic agents active in MS [7,12].

The cuprizone mouse model and experimental autoimmune encephalomyelitis (EAE) are the most commonly used animal models to study active compounds designed to alleviate MS pathophysiological processes. EAE is induced by immunization with myelin peptides or CNS tissue and an immune-activating adjuvant (containing bacterial components). This generates key MS pathological features, such as inflammatory infiltrates, axonal loss, demyelination and gliosis [13]. The other model is based on intoxication with the copper chelating agent cuprizone [14,15]. The cuprizone-induced neurotoxicity is caused by enzyme inhibition and copper homeostasis disruption in the CNS, leading to neuroinflammation, oxidative stress, myelin sheath degeneration, oligodendrocyte apoptosis, disturbance of neurotransmitter metabolism, axonal beading, microgliosis and astrogliosis [16]. Furthermore, the demyelinating lesions in intoxicated mice were linked with impaired motor coordination and spatial memory, decreased social interaction and increased exploratory behavior [14,17].

Astrocytic TRPA1 (transient receptor potential ankyrin 1) calcium channels regulate oligodendrocyte apoptosis, indicating that pharmacologically inhibiting TRPA1 might exhibit protective effects against demyelination in the murine cuprizone model [18]. TRPA1, a member of the transient receptor potential (TRP) superfamily, primarily acts as a sensor for reactive chemical species. It is expressed in various tissues, including rodent hippocampal astrocytes, hence regulating calcium levels, synaptic transmission and long-term potentiation, but also in the hippocampal and cortical neurons, cortical vessels, supraoptic nucleus, striatum, amygdala, olfactory bulb, brain stem and spinal cord [19,20,21,22,23,24]. TRPA1 is activated endogenously by oxidized and nitrated lipids, small reactive oxygen species, 7-dehydrocholesterol and its inhibition by small-molecule antagonists could offer novel means for treating neuropathic pain inflammation and multiple sclerosis [17,25,26,27,28,29].

We previously implemented an artificial intelligence algorithm [30] and an in silico screening algorithm as drug repurposing tools for discovering new potential therapeutic solutions for MS, predicting that the antidepressant desvenlafaxine, antipsychotic paliperidone and gout medication febuxostat might act as TRPA1 antagonists [31]. We herein investigated the interaction between the proposed molecules and TRPA1 calcium channel activity, and the effects of febuxostat and its parent drugs venlafaxine and risperidone on cuprizone-induced acute demyelination in mice, by evaluating the locomotor activity, motor performance, sensitivity to a cold stimulus, myelin integrity and oxidative stress markers. Since both desvenlafaxine and paliperidone are metabolites with high treatment costs and suffer from important safety concerns, we chose to investigate in vivo the effects of their parent molecules [32,33].

Venlafaxine is a serotonin-norepinephrine reuptake inhibitor used in the treatment of depression, anxiety disorders and neuropathic pain [34,35,36]. Moreover, venlafaxine was found to decrease the levels of pro-inflammatory cytokines [37,38], while the major metabolite desvenlafaxine prevented stress-induced white matter injury [39]. Risperidone is an atypical antipsychotic used to manage schizophrenia, bipolar disorder and autism-related irritability [40]; previous studies report that it also ameliorates neuroinflammation [41,42]. Febuxostat is a long-term therapeutic option for gout, acting as a non-competitive xanthine oxidase inhibitor [43]. The urate-lowering agent has been previously shown to inhibit the inflammatory response in astrocytes [44].

## 2. Results

### 2.1. Behavioral Tests

#### 2.1.1. Locomotor Activity

**Figure 1:**
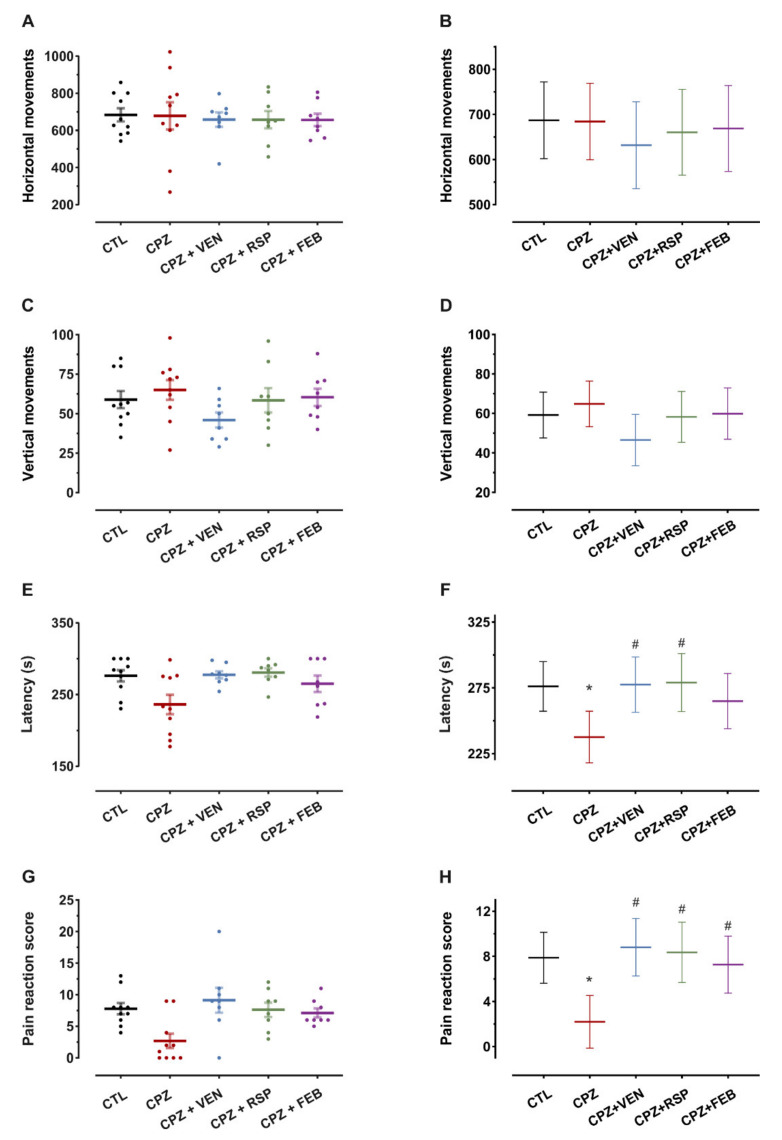
Behavioral tests following 5-weeks treatment. (**A**) Spontaneous locomotor activity—mean ± S.E.M (standard error of the mean) of total number of horizontal movements. (**B**) Mean and 95% confidence interval estimates of the horizontal movements after treatment. (**C**) Spontaneous locomotor activity—mean ± S.E.M of total number of vertical movements. (**D**) Mean and 95% confidence interval estimates of the vertical movements after treatment. (**E**) Motor coordination— mean ± S.E.M of latency (s) of falls off the rotating drum. (**F**) Mean and 95% confidence interval estimates of the fall latencies after treatment. (**G**) Cold temperature sensitivity— mean ± S.E.M of total pain reaction score. (**H**) Mean and 95% confidence interval estimates of the pain reaction score after treatment. CTL—control (*n* = 10); CPZ—cuprizone 400 mg·kg^−1^ (*n* = 10); CPZ + VEN—cuprizone 400 mg·kg^−1^ and venlafaxine 60 mg·kg^−1^ (*n* = 8); CPZ + RSP—cuprizone 400 mg·kg^−1^ and risperidone 2 mg·kg^−1^ (*n* = 8); CPZ + FEB—cuprizone 400 mg·kg^−1^ and febuxostat 5 mg·kg^−1^ (*n* = 8). Baseline measurements for the corresponding tests before treatment were used as covariates for estimation in panels B, D, F and H. * *p* < 0.05 vs. CTL; ^#^ *p* < 0.05 vs. CPZ.

Locomotor activity following the 5-week cuprizone (CPZ) and drug treatments was investigated, and baseline measurements served as a covariate (univariate ANCOVA). No significant alterations were observed in the total number of horizontal movements within the tested groups (F_(4,38)_ = 0.24, *p* = 0.92, Figure 1A,B). Similarly, the number of vertical movements was not different (univariate ANCOVA, F_(4,38)_ = 1.2, *p* = 0.33, Figure 1C,D).

#### 2.1.2. Motor Function

After 5 weeks of cuprizone treatment, significant motor treatment effects could be observed, measured by the fall latency on the rotarod (univariate ANCOVA with baseline as a covariate, F_(4,38)_ = 3.0, *p* = 0.03, Figure 1E,F). Cuprizone treatment significantly reduced the fall latency of mice by 15% compared to the control (CTL) group (*p* = 0.007, prespecified comparison). Venlafaxine (VEN) treatment provided a protective effect against the action of cuprizone. In the CPZ + VEN group, the latency was increased by 18% compared to the CPZ group (*p* = 0.008, Bonferroni-Holm correction). Risperidone also showed a protective effect, increasing the latency by 19% compared to cuprizone (*p* = 0.009, Bonferroni-Holm correction). Febuxostat treatment did not significantly increase the fall latency (*p* = 0.07, Bonferroni-Holm correction).

#### 2.1.3. Cold Sensitivity

Pain reaction scores were significantly affected by the treatments (univariate ANCOVA) and baseline measurements were used as covariates (F_(4,38)_ = 5.1, *p* = 0.002). Cuprizone lowered the pain reaction score by 65% in mice treated for five weeks (*p* = 0.002, prespecified comparison). Venlafaxine, risperidone and febuxostat treatment groups exhibited higher pain reaction scores when compared to the CPZ group (3.4-fold, *p* < 0.001 for venlafaxine, 2.8-fold, *p* = 0.002 for risperidone and 2.6-fold, *p* = 0.006 for febuxostat, Bonferroni-Holm corrected significance levels, Figure 1G,H).

### 2.2. Demyelination in the Corpus Callosum (CC)

**Figure 2:**
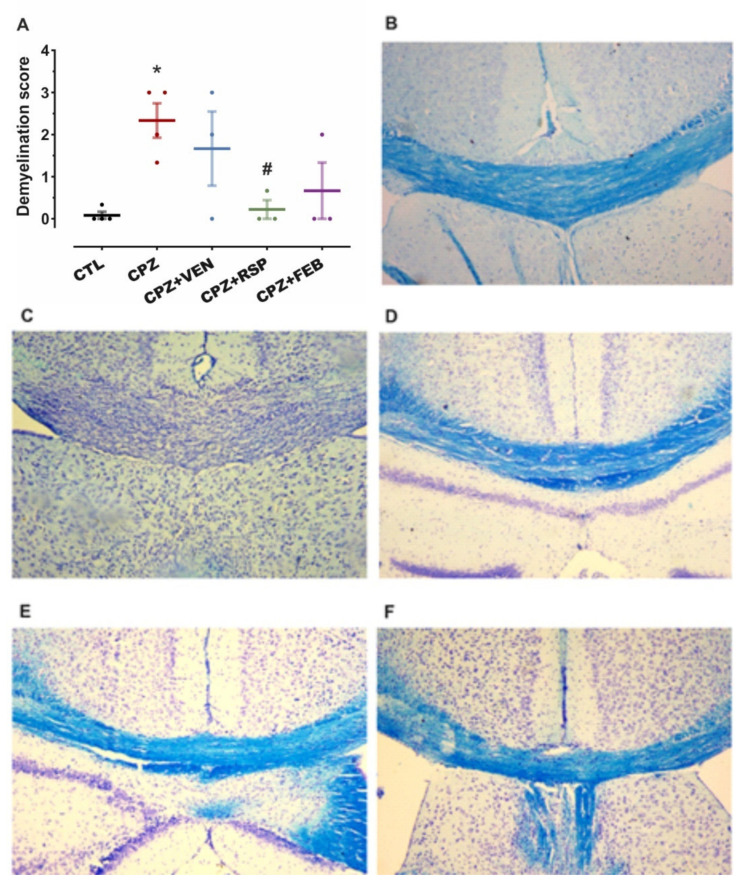
Myelin integrity in mice *corpus callosum* (CC) after 5 weeks of treatment; staining with Luxol Fast Blue (LFB) and cresyl violet (CV). (**A**) Variation in demyelination score between treated groups. (**B**) CTL, (**C**) CPZ, (**D**) CPZ + VEN, (**E**) CPZ + RSP, (**F**) CPZ + FEB. Data are presented as means ± standard error mean (S.E.M). CTL—control (*n* = 4); CPZ—cuprizone 400 mg·kg^−1^ (*n* = 4); CPZ + VEN—cuprizone 400 mg·kg^−1^ and venlafaxine 60 mg·kg^−1^ (*n* = 3); CPZ + RSP—cuprizone 400 mg·kg^−1^ and risperidone 2 mg·kg^−1^ (*n* = 3); CPZ + FEB—cuprizone 400 mg·kg^−1^ and febuxostat 5 mg·kg^−1^ (*n* = 3). * *p* < 0.05 vs. CTL; ^#^ *p* < 0.05 vs. CPZ.

Myelin integrity was determined with Luxol fast blue (LFB), and cresyl violet (CV) staining of the mice’s brain tissue. Analysis of variance showed a main effect of the treatment on demyelination scores (F_(4,12)_ = 4.3, *p* = 0.02). Cuprizone intoxication for 5 weeks yielded significant demyelination in the mice brain CC (*p* = 0.004 vs. CTL). Although myelin degradation was nominally lower after all three administered drugs (Figure 2B–F), this was only significant for risperidone (91% reduction, *p* = 0.01 vs. CPZ, Bonferroni-Holm corrected post-hoc test, Figure 2A).

### 2.3. Biochemical Assay of Mouse Brain Homogenates

The extent of oxidative stress was investigated in mice brain tissue samples following the 5-week cuprizone treatment by determining the susceptibility to lipid peroxidation of the mitochondrial membranes, superoxide dismutase (SOD) activity, the neuronal nitric oxide synthase (nNOS) activity and total thiols content.

**Figure 3:**
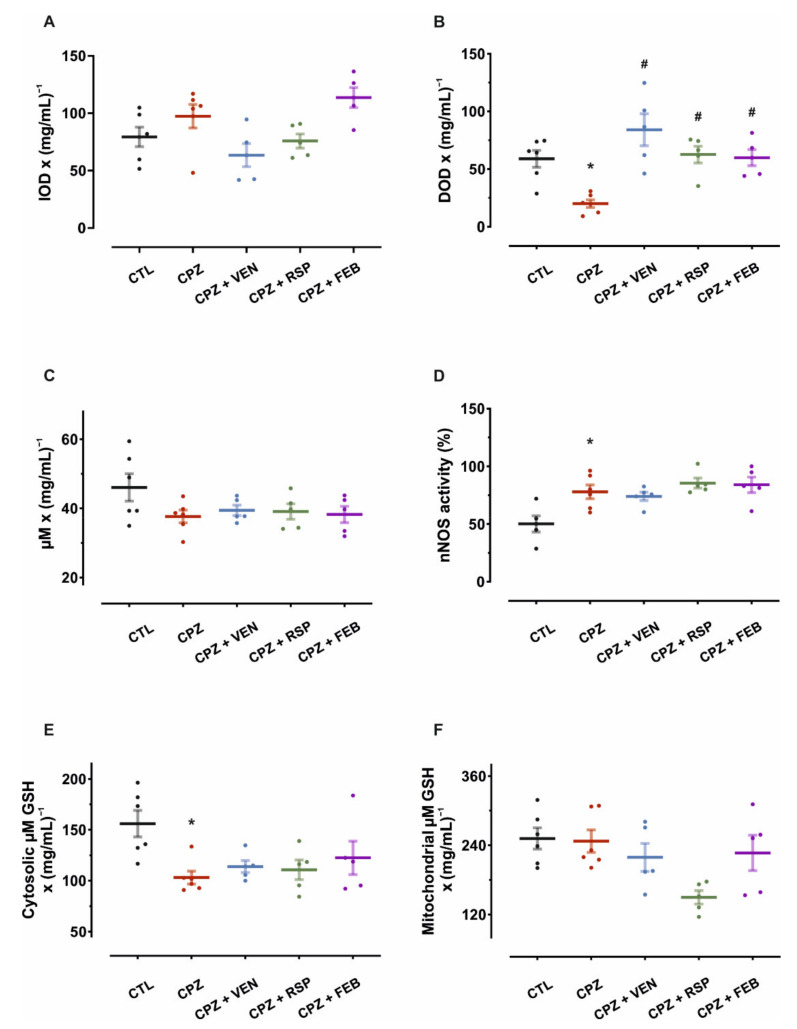
Results from biochemical assays of mouse brain homogenates. (**A**) Susceptibility to lipid peroxidation of the mitochondrial membrane, determined with DPPP method—percentage increase of optical density (IOD) reported to protein content, after 5 min. (**B**) Effects of 5-week treatments on mouse brain SOD activity—percentage decrease in optical density (DOD) reported to protein content. (**C**) Variation of total nitrites in mouse brain tissues—modified Griess method. No significant variations of total nitrites were observed between groups. (**D**) nNOS activity (percentage increase of total nitrites when nNOS activity is assessed). (**E**) Cerebral cytosolic total thiols concentrations, expressed as glutathione (GSH) equivalents to protein ratio. (**F**) Mitochondrial total thiols. Data are presented as means ± S.E.M. CTL—control (*n* = 6); CPZ—cuprizone 400 mg·kg^−1^ (*n* = 6); CPZ + VEN—cuprizone 400 mg·kg^−1^ and venlafaxine 60 mg·kg^−1^ (*n* = 5); CPZ + RSP—cuprizone 400 mg·kg^−1^ and risperidone 2 mg·kg^−1^ (*n* = 5); CPZ + FEB—cuprizone 400 mg·kg^−1^ and febuxostat 5 mg·kg^−1^ (*n* = 5). * *p* < 0.05 vs. CTL; ^#^ *p* < 0.05 vs. CPZ.

Regarding the susceptibility to lipid peroxidation of the mitochondrial membranes, the results of the diphenyl-1-pyrenylphosphine (DPPP) method showed significant group differences (one-way ANOVA, F_(4,22)_ = 4.5, *p* = 0.008). Mice from the CPZ group were not different in peroxidation susceptibility to control (*p* = 0.15, Figure 3A), such that further comparisons were not performed.

The assessment of brain SOD activity revealed a significant treatment effect on enzyme activity (one-way ANOVA, F_(4,22)_ = 8.4, *p* < 0.001). Following cuprizone-induced demyelination, SOD activity decreased by 66% compared to the control (*p* = 0.002). Compared to cuprizone alone, SOD activity was increased 4.2-fold for venlafaxine (*p* < 0.001), 3.1-fold for risperidone (*p* = 0.001) and 3.0-fold for febuxostat-treated mice (*p* = 0.002, Bonferroni-Holm corrected post-hoc tests, Figure 3B).

Total nitrites and nNOS activity were assessed in mouse brain tissues after the 5-week treatment period using Griess modified method. All groups that were subjected to cuprizone-induced demyelination showed only nominally modified levels of total nitrites (one-way ANOVA, F_(4,22)_ = 1.8, *p* = 0.16, Figure 3C). Contrastingly, one-way ANOVA revealed a significant treatment effect on nNOS (F_(4,22)_ = 6.0, *p* = 0.002). Administration of cuprizone increased the nNOS activity by 56% (*p* = 0.002, vs. CTL, prespecified comparison), while co-treatment with venlafaxine, risperidone and febuxostat did not alter this (*p* = 0.62, *p* = 0.36 and *p* = 0.45, respectively, in comparison to the CPZ group, Bonferroni-Holm corrected post-hoc tests, Figure 3D).

Brain cytosolic and mitochondrial total thiol concentrations were also investigated. One-way ANOVA revealed a significant treatment effect on cytosolic thiol concentrations (F_(4,22)_ = 3.9, *p* = 0.02). These levels were lower for cuprizone-treated animals compared to the control (34%, *p* = 0.002), cotreatment with venlafaxine, risperidone and febuxostat did not alter this (*p* = 0.49, *p* = 0.63 and *p* = 0.22, Bonferroni-Holm corrected post-hoc tests, Figure 3E). A significant treatment effect was detected on mitochondrial total thiols (one-way ANOVA, F_(4,22)_ = 3.5, *p* = 0.02). However, no significant differences were observed between the control and CPZ groups regarding the mitochondrial total thiol concentrations (*p* = 0.88), and further comparisons were not performed (Figure 3F).

### 2.4. Functional Assay of Human TRPA1 Expressing Cells

**Figure 4:**
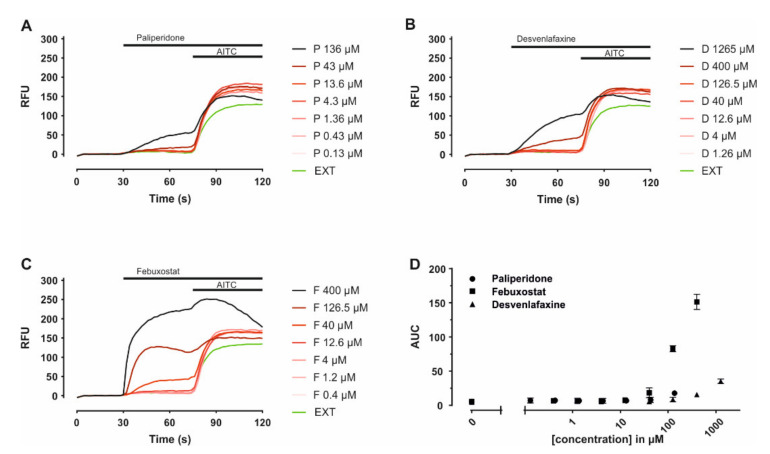
Paliperidone, desvenlafaxine and febuxostat effects in hTRPA1 expressing HEK293T cells. (**A**–**C**) Individual time courses of fluorescence displayed as means of 3 replicates for each given concentration. (**D**) Area under the curve calculated for the interval 30–70 s, reflecting the addition of the compounds until the addition of AITC.

**Figure 5:**
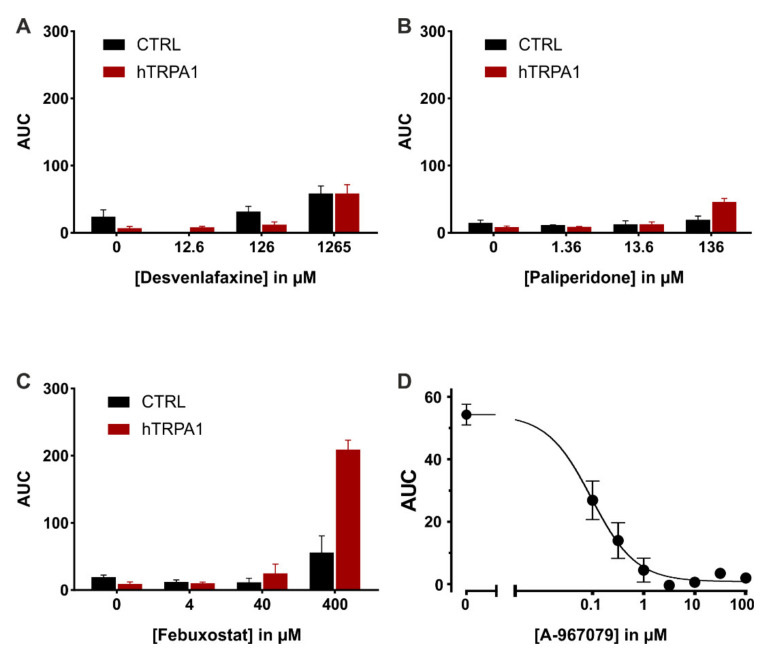
Direct effects of paliperidone, febuxostat and desvenlafaxine in hTRPA1-transfected compared to non-transfected HEK293T cells. (**A**–**C**) Areas under the curve calculated for the application period of each compound, in hTRPA1 expressing HEK293T cells (in red), and in untransfected cells (in black). Data are displayed as the mean of four replicates each ± S.E.M. (**D**) Three-parameter logistic fit of the inhibitory effect of the TRPA1 antagonist A-967079 against febuxostat 126 µM. Data are displayed as mean ± S.E.M. of 6 replicates each.

Venlafaxine is extensively metabolized to desvenlafaxine via CYP_2_D_6_, which is also used as a stand-alone treatment for major depressive disorders [45], while paliperidone is the major active metabolite of risperidone [46]. Febuxostat is metabolized via cytochrome P450 enzymes to three active metabolites; thus, the parent drug was further investigated. To address the effects of paliperidone, febuxostat and desvenlafaxine on TRPA1, these were probed in heterologous expression, in hTRPA1 expressing HEK293T cells, by means of a medium-throughput assay. In each experiment, the substance of interest was applied before the established TRPA1 agonist allyl isothiocyanate (AITC), allowing to estimate whether they function as antagonists or agonists. All three compounds seemed to trigger intracellular calcium level increases at higher concentrations, in particular for febuxostat (Figure 4). Compared to control experiments a significant fluorescence increase was observed for febuxostat 126 µM (*p* < 0.001) and concentrations above; this also applies for desvenlafaxine 1265 µM (*p* < 0.001), as well as paliperidone 136 µM (*p* = 0.01). The agonistic effects were further probed in hTRPA1 expressing HEK293T cells in a comparative manner to untransfected cells, serving as control. The increase in intracellular calcium was similar throughout the concentration range both in transfected and untransfected cells for paliperidone and desvenlafaxine (Figure 5A,B). However, febuxostat elicited a TRPA1-dependent calcium increase at higher concentrations (Figure 5C). Responses to febuxostat 126 µM were inhibited by the TRPA1 antagonist A-967079 in a dose-dependent manner with an IC50 of 0.09 µM (0.05 to 0.14, 95% C.I.) (Figure 5D).

## 3. Discussion

The cuprizone mouse model of acute demyelination is one of the established animal models for demyelination/remyelination used in the pursuit of discovering novel therapeutics for MS and other demyelinating diseases [47]. Toxic demyelination can be induced by oral administration of a daily dose of 400 mg·kg^−1^ cuprizone or by feeding the rodent the toxic compound mixed with ground chow in 0.2% concentration over a 5–6 week period [15,47]. It was previously illustrated that cuprizone selectively affects mature oligodendrocytes (OLGs) and oxidative stress is a direct cause of apoptosis. Some of the redox balance disturbances that increase the vulnerability of mature OLGs during cuprizone treatment are reduction in glutathione (GSH) content, increase in lipid peroxidation, decreased activity of SOD and catalase (CAT) [16]. Increased activity of nNOS was also correlated with cuprizone-induced demyelination in the CNS [48]. Demyelination occurs mainly in the *corpus callosum* (CC) and the cerebral cortex; loss of myelin is usually quantified by LFB or immunohistochemical staining or by electron microscopy and magnetic resonance imaging. The behavioral traits of demyelinated mice are well correlated with the extent of white matter degradation and include increased locomotor activity and mild impairment of motor function, while no alterations in sensitivity to nociception were noticed [14,17,49,50].

The aim of our study was to investigate the possibility to repurposing three drugs using cuprizone-induced acute demyelination as a model for demyelinating diseases, such as MS. In our experiment, cuprizone-treated mice showed significant demyelination in CC, impaired motor performance and a decrease in sensitivity to cold nociceptive stimuli. No significant effects were observed on exploratory behavior. Furthermore, cuprizone administration for five weeks led to an elevation of nNOS activity, and reductions of SOD activity and cytosolic GSH levels, without altering the peroxidation of the mitochondrial membrane.

Venlafaxine treatment with a daily dose of 60 mg·kg^−1^ offered the least protection against demyelination of the *corpus callosum* among the three administered drugs. Mice treated with the antidepressant showed a mild decrease in vertical movements and prevented the motor performance impairment and hyposensitivity to cold temperature induced by cuprizone. Venlafaxine successfully prevented the decrease of SOD activity observed in demyelinated mice. Its main metabolite, desvenlafaxine, did not show antagonistic effects on TRPA1 transfected cells; thus, the therapeutic effects of venlafaxine seem to be unrelated to astrocytic TRPA1 inhibition. Previous studies reported that venlafaxine (6, 20 and 60 mg·kg^−1^) reduced neuroinflammation in the murine experimental model of autoimmune encephalomyelitis by suppressing pro-inflammatory cytokines and that it mediates immunomodulatory mechanisms in the CNS, hence reducing neuroinflammation [51,52]. Venlafaxine (16 mg·kg^−1^) administered intraperitoneally to mice produced significant anti-apoptotic effects by activating the Akt pathway in the hippocampus [53].

The antipsychotic risperidone showed the most protection against demyelination when compared to the cuprizone group. Risperidone (2 mg·kg^−1^) prevented motor impairment and cold hyposensitivity induced by cuprizone. Risperidone treatment led to normal levels of SOD activity but failed to ameliorate the cuprizone-induced increase of nNOS activity. Mice that received cuprizone and risperidone showed an apparent decrease in mitochondrial thiol content, which is in line with previous reports detailing its detrimental effects on the mitochondria [54,55]. Paliperidone did not influence TRPA1 calcium channel activity; thus, other mechanisms are responsible for the beneficial effects of risperidone. One study showed that risperidone (3 mg·kg^−1^) reduced microglia and macrophage activation in an EAE model, but a recent clinical trial indicated that progressive multiple sclerosis patients may experience increased sensitivity to risperidone [56,57].

The myelin sheath integrity of mice who received febuxostat (5 mg·kg^−1^) was not significantly different from cuprizone. Motor performance was not significantly improved in comparison to the cuprizone group. However, sensitivity to cold stimuli was significantly higher in comparison with the cuprizone-treated mice. Co-administration of cuprizone and febuxostat did not significantly change nNOS activity, which was similar to cuprizone-treated mice, while the activity of SOD was significantly higher when compared to demyelinated mice. Interestingly, febuxostat showed agonistic activity on TRPA1 channel at high concentrations (126 µM), but this effect is not relevant for its capacity to alleviate cuprizone-induced demyelination. It is unclear if such activity has detrimental effects on myelin integrity since pharmacological activation of TRPA1 was not studied in relation to demyelination so far. Moreover, the maximum plasma concentration of febuxostat in mice after oral administration of 150 mg·kg^−1^ was 4.1 µM, which is below the level of TRPA1 activation [58]. Inhibition of xanthine oxidase by febuxostat (0.75 mg·kg^−1^) was shown to prevent axon and myelin loss and restore mitochondrial energy production in EAE [59,60].

The lack of significant antagonistic activities on TRPA1 highlighted that the tested drugs were false positives in our previous in silico study. On the other hand, febuxostat was found to activate TRPA1 calcium channels, implying that the screening method might also identify possible agonists. Considering the in vitro results, the methodology used for discovering possible TRPA1 antagonists could be further improved to obtain a lower false-positive rate and better discrimination between agonists and antagonists. The purpose of the in vitro study was to confirm or deny the hypothesis that the tested drugs would ameliorate cuprizone-induced demyelination and its detrimental effects via TRPA1 inhibition, alongside other possible molecular mechanisms. Our findings suggest that the protective effects of venlafaxine, risperidone and febuxostat are obtained in a TRPA1-independent manner, probably through modulatory effects on inflammation pathways. For instance, previously published animal studies revolving around inflammation showed that venlafaxine, risperidone and febuxostat reduced inflammatory responses by lowering the levels of astrocytic and microglial pro-inflammatory cytokines (TNF-α, IL-1β and IL-6) [37,41,42,44], which are also involved in the pathology of cuprizone model of demyelination [16].

The limitations of the present study include the low number of samples that were available for histological and biochemical assays, thus decreasing the power of statistical tests to identify significant differences between groups. Moreover, no effects of the studied drugs on brain cytokine levels were assessed in our study. Another limitation is represented by the investigation of a single dosage for each drug, and dose-dependent effects could be further studied.

One final limitation consisted of the fact that pharmacotherapy for demyelinating diseases is initialized after the onset and diagnosis of the respective pathology, while in the murine model of demyelination, treatments were administered since the first day of cuprizone intoxication. Past investigations reported that acute demyelination and neuroinflammation occur in the cuprizone mouse model within a few days to three weeks of daily intoxication, the maximum damage appears after five weeks, following a remyelination phase due to proliferation and differentiation of oligodendrocyte precursor cells, which becomes apparent after six weeks [14,16]. Even though the behavioral deficits were ameliorated, it is unclear whether the studied drugs prevented or treated the manifestations of cuprizone-induced demyelination since some of the biochemical disturbances were not significantly reversed (i.e., increased activity of nNOS, which serves as a marker for cuprizone intoxication [48]). Therefore, the proposed drugs could be used as therapeutic options for preventing further damage in the CNS, rather than promoting remyelination, since this effect was not addressed in our study.

Our findings indicate that all three candidates could be suitable for treating demyelinating diseases, considering the beneficial effects shown on behavioral tests, myelin integrity and biochemical markers of cuprizone intoxication. Further studies are needed to evaluate the feasibility of such drugs as treatments for MS patients.

## 4. Materials and Methods

### 4.1. Animals and Treatments

Experimental procedures were carried out in compliance with bioethics norms proposed by the NIH Guide for the Care and Use of Laboratory Animals. The experimental protocol was approved by the Bioethics Commission of the Faculty of Pharmacy, University of Medicine and Pharmacy Carol Davila, Bucharest, Romania (ANAI02/01.02.2020).

Female C57BL6/J mice (8–12 weeks old) were acquired from INCDMI Cantacuzino (Cantacuzino National Institute of Research, Bucharest, Romania) and were housed in plexiglass cages at a 12 h/12 h light-dark cycle, under constant humidity (35–45%) and temperature (20–22 °C), monitored with a thermohygrometer. Food (rodent ground chow, INCDMI Cantacuzino, Bucharest, Romania) and drinking water were available to the animals ad libitum.

Animals were left to acclimatize with the new habitat for one week, were thereafter divided into five experimental groups and body weights were measured every two days. All treatments were administered daily for five weeks by oral gavage as follows: distilled water for control group (CTL, *n* = 10), cuprizone 400 mg·kg^−1^ and distilled water for the disease model group (CPZ, *n* = 10), cuprizone and venlafaxine 60 mg·kg^−1^ (CPZ + VEN, *n* = 8), cuprizone and risperidone 2 mg·kg^−1^ (CPZ + RSP, *n* = 8), and cuprizone and febuxostat 5 mg·kg^−1^ (CPZ + FEB, *n* = 8) as drug treatment evaluation groups. Drug treatments were administered since the first day of the experiment, together with cuprizone. After five weeks, all groups were subjected to behavioral tests in order to investigate the efficacy of administered drugs in preventing functional alterations induced by acute demyelination. At the end of the experiment, mice were euthanized for further investigations in accordance with the standard guidelines by intraperitoneal injection with 200 mg·kg^−1^ of thiopental sodium. Subsequently, each group was divided into two subgroups for histological and biochemical assays.

The number of animals per group was established considering both bioethics norms and experimental protocols reported in previous studies [15,60,61,62,63]. The doses of administered drugs were chosen according to published literature, relatively high doses being used since the proposed drugs were not investigated in close relation with their main therapeutic indications [51,56,64].

### 4.2. Behavioral Tests

Behavioral tests were carried out for evaluating the spontaneous locomotor activity, motor function deficits and sensitivity to a cold stimulus. Previous studies reported that mice intoxicated with cuprizone did not show signs of thermal or mechanical hyperalgesia [49]. In our study, we investigated if white matter deficits would alter cold sensitivity in cuprizone-treated mice and the capability of administered drugs to prevent such effects.

#### 4.2.1. Locomotor Activity

The locomotor activity was assessed using a system composed of a cage with transparent plexiglass walls (40 × 40 × 25 cm), equipped with two sets of photo beams (Ugo Basile, Gemonio, VA, Italy). Mice were placed individually in the apparatus and the total photo-beam interruptions were measured for 5 min, corresponding to total horizontal and vertical movements performed by each animal [65,66].

#### 4.2.2. Motor Function

The motor performance was evaluated using the Rotarod apparatus (Ugo Basile, Gemonio, VA, Italy). Each mouse was subjected to a training procedure prior to the testing day, identical to the testing protocol. The motor coordination assessment protocol consisted of three trials of testing with a 15 min interval between trials. Each mouse was placed in the apparatus and the rotating drum was set to accelerate from 4 to 40 revolutions per minute (rpm) over a 5 min period. The latency of the first fall off the drum was measured [49,67].

#### 4.2.3. Cold Sensitivity

The sensitivity to a cold stimulus was evaluated using the acetone evaporation test.

Mice were left to acclimatize for 1 h in transparent plexiglass cages, provided with a floor made of a wire mesh (Hargreaves apparatus, Ugo Basile, Gemonio, VA, Italy). A drop of acetone was dabbed onto the plantar surface of the hindpaws with a syringe, its evaporation producing cold, thermal nociception [68]. The acetone drop was applied three times to each hind paw, alternately at a 5 min interval between applications. After the application of the stimulus, the nociceptive behavior was determined for 50 s, and the measurements were performed after a period of 10 s, corresponding to the physiological reactions. The pain sensitivity of mice was assessed by measuring two parameters: total reaction time and a total score of pain reactions, resulting from the sum of the measurements from the six determinations for each mouse. The total score was determined by assigning an individual score to each type of reaction and calculating their sum as follows: no response (0), brief paw lift, paw sniff or limping (1), jumping or paw shaking (2), multiple paw lifts or licks (3), prolonged paw lifting, shaking or licking (4) [69,70].

### 4.3. Histological Staining

In our experiment, Luxol fast blue (LFB) staining and cresyl violet (CV) counterstaining were used to evaluate the demyelinating lesions in the *corpus callosum* (CC). Mice from histological assay subgroups were perfused intracardially with 10% formaldehyde in phosphate-buffered saline (PBS). Mouse brains were harvested and were cut in coronal slices between levels 1 and −1 mm from bregma and were fixed in 10% formaldehyde-PBS for 72 h. The fixed brains were further processed by following a paraffin-embedding protocol consisting of several dehydration steps by multiple washes in xylene and alcohol solutions. The dehydrated specimens were thereafter embedded in paraffin blocks and 10 µm thick sections were made using a microtome (Microm HM 310, LabX, Midland, ON, Canada). Brain sections were mounted on glass slides and were subjected to deparaffinization and rehydration, LFB staining, lithium carbonate differentiation and CV counterstaining, followed by dehydration and application of the mounting agent. Images of the brain sections were acquired using a light microscope (Euromex bScope, Euromex Microscopen BV, Papenkamp, Netherlands) and digital camera (C-mount camera, Carl Roth GmbH + Co. KG, Karlsruhe, Germany). The severity of demyelination was assessed using a 0–3 point semiquantitative scale as described by other studies: no demyelination (0), rare focal demyelination (1), multiple focal demyelination (2), large areas of demyelination (3) [71].

### 4.4. Biochemical Assays

Mouse brains were harvested from the biochemical assay subgroups after euthanasia in order to evaluate the redox status after cuprizone intoxication. Tissue homogenates were obtained by using a 1:10 ratio (*w*/*v*) between tissue and sucrose 0.25 M and homogenized with a RW 14 basic homogenizer (IKA, Königswinter, Germany). Mitochondria were separated from tissue homogenates taking into consideration the previously described methods [72,73], in 3 consecutive centrifugation steps at 600× *g* · 4 °C, 10,000× *g* · 4 °C and, respectively, 5000× *g* · 4 °C (Z 326 K centrifuge, HERMLE Labortechnik, Wehingen, Germany), being thereafter suspended in PBS in a 1:1 ratio (*v*/*v*) to the initial homogenate volume. Tissue homogenates and mitochondrial preparations were diluted 1:10 with PBS prior to analysis.

#### 4.4.1. Mitochondrial Membrane Lipid Peroxidation

The susceptibility to lipid peroxidation of the mitochondrial membrane was assessed with diphenyl-1-pyrenylphosphine (DPPP, ThermoFisher Scientific, Waltham, MA, USA) according to the procedure described by Margină et al. [74,75]. Samples (isolated brain mitochondria) were incubated with DPPP 100 µM (10:1 ratio) for 20 min at room temperature (RT) for its accumulation in membranes, followed by the addition of cumene hydroperoxide (CHP) 1 µM for the induction of lipid peroxidation. Membrane lipid peroxidation was monitored for 5 min at λ_excitation_ = 350 nm/λ_emmision_ = 380 nm on a PerkinElmer spectrofluorometer (Waltham, MA, USA) and compared to baseline absorbance before adding CHP. Results are reported as percentage increase of OD (IOD, expressed in RFU) at 5 min versus baseline: IOD = 100 × (OD_T=5_ − OD_T=0_)/OD_T=0_. Also, the recorded absorbance to protein content ratio was calculated for each read.

#### 4.4.2. Brain Superoxide Dismutase Activity

The activity of SOD was assessed with a colorimetric activity kit (cat. no. 19160, Sigma Aldrich, St. Louis, MO, USA), employing a xanthine oxidase superoxide anion generating system that yields a colored formazan, detectable at 440 nm. In the presence of samples containing SOD, the decrease in OD (DOD) is directly proportional to the enzyme activity. The experimental procedures were in accordance with the manufacturer’s instructions, results being expressed as percentage DOD reported to protein content.

#### 4.4.3. Griess Assessment of nNOS Activity

We determined total nitrites (after the reduction of nitrates), as the end products of neuronal nitric oxide synthase (nNOS), with the previously employed Griess method [76,77] modified using vanadium (III) instead of cadmium for the reduction of nitrates to nitrites [78].

For the assessment of total nitrites, the sample (50 µL) was treated with vanadium (III) chloride 0.8% in HCl 1M (50 µL) and Griess modified reagent 4% (100 µL), incubated at RT for 30 min and OD measured at 540 nm. A standard curve of NaNO_2_ was measured for each experiment and results are reported as µM NO_2_^−^ to protein ratio.

Further, we determined the activity of nNOS incubating tissue homogenates samples (50 µL) with arginine 0.013% (10 µL), FAD (10 µL) and NADPH2 (50 µL) for 60 min · 37 °C, followed by the addition of VCl_3_ 0.8% in HCl 1M (50 µL) and Griess modified reagent (100 µL), reading at 540 nm after 30 min at RT, versus a sample blank without enzyme substrate and cofactors. nNOS activity is expressed as the % increase of total nitrites = 100 × (DO_sample_ − DO_sample blank_)/DO_sample blank_.

#### 4.4.4. Assessment of Total Thiols

Total thiols were assessed using a previously described method [79], using a 1:4 ratio between sample (tissue homogenate/mitochondrial preparation) and Ellman reagent. Results are expressed as glutathione (GSH) equivalents (µM) to protein ratio (mg·mL^−1^).

#### 4.4.5. Protein Content

For the assessment of total protein content in tissue homogenates and mitochondrial preparations, we employed the well-established Lowry method [80], with a standard curve of bovine serum albumin up to 1.5 mg·mL^−1^.

### 4.5. TRPA1 Activity Assay

For fluorescent imaging plate reader calcium assays, non-transfected and transfected HEK293T cells (which are widely used for establishing detailed pharmacological and biophysical profiles for drugs and their targets, including experiments involving TRPA1 function [81,82,83]) were seeded at a density of 30,000 cells/well in a black flat-bottom 96-well cell culture plate (Greiner Bio-One, Kremsmünster, Austria) coated with poly-d-lysine (100 mg·mL^−1^, Sigma-Aldrich, St. Louis, MO, USA). Then they were loaded with the Calcium 6 dye for two hours (Calcium 6 Kit by Molecular Devices, San Jose, CA, USA) in an extracellular solution. According to the manufacturer’s protocol, cells were not washed, but extracellular dye was chemically quenched. Calcium 6 fluorescence excited at 488 nm every 2 s served as an index of intracellular calcium. Assays were carried out at 25 °C with a fluorescent imaging plate reader with an integrated pipettor (FlexStation 3, Molecular devices, San Jose, CA, USA). A volume of 50 µL containing test substances was added automatically according to a preset protocol into 100 µL of extracellular solution in the wells, 30 s after the start of the measurement for the compounds of interest (desvenlafaxine, paliperidone and febuxostat) and 70 s for AITC.

The drug concentrations for the dose-response were chosen such that at the upper end of the scale, the applied solution is delivered together with no more than 1% of solvent (DMSO) based on the solubility limit of the stock solutions to exclude solvent driven effects.

### 4.6. Reagents

Cuprizone (bis-cyclohexanone oxaldihydrazone) was synthesized in our lab starting from oxalic acid (Chemical Company, Iași, Romania), methanol, hydrazine hydrate, and cyclohexanone (all from Sigma Aldrich, St. Louis, MO, USA). Cuprizone was purified by recrystallization from isopropanol and its purity was validated using ^1^H-NMR and ^13^C-NMR spectra (Gemini 300 BB Varian, Palo Alto, CA, USA), infrared spectra (FT/IR-4200, JASCO, Tokyo, Japan), and elemental analysis (PerkinElmer 2400 Series II CHNS/O Elemental Analyzer, Waltham, MA, USA).

Venlafaxine (KRKA D.D., Novo Mesto, Slovenia), risperidone (Janssen Pharmaceutica N.V., Beerse, Belgium) and febuxostat (Tokyo Chemical Industry CO., LTD., Tokyo, Japan) were administered as aqueous solutions or suspensions. Thiopental sodium, LFB, CV, lithium carbonate, cumene hydroperoxide and formaldehyde were acquired from Sigma-Aldrich (St. Louis, MO, USA). Desvenlafaxine was acquired from ChemPur (CHEMPUR Feinchemikalien und Forschungsbedarf GmbH, Karlsruhe, Germany) and paliperidone from TCI (Tokyo Chemical Industry CO., LTD., Tokyo, Japan).

For the preparation of tissue homogenates and mitochondrial preparations, a 0.25 M sucrose (Sigma-Aldrich, St. Louis, MO, USA) solution was used. PBS (Biochrom AG, Germany) was used for sample dilution.

CuSO_4_ (Chemical Company, Iași, Romania) 0.5%, Na_2_CO_3_ 2% (Chemical Company, Iași, Romania) in NaOH 0.1 M (Chemical Company, Iași, Romania), Folin-Ciocalteu reagent (Merck, Kenilworth, NJ, USA) were employed for protein assessment, with bovine serum albumin (Sigma-Aldrich, St. Louis, MO, USA) used for a standard curve.

Griess modified reagent, vanadium (III) chloride, were purchased from Sigma Aldrich (St. Louis, MO, USA), FAD, NADPH2 and arginine from Merck Millipore, Burlington, MA, USA, and DPPP from ThermoFisher Scientific (Waltham, MA, USA).

The extracellular solution used for cellular experiments contains (in mM): 145 NaCl, 5 KCl, 10 glucose, 10 4-(2-hydroxyethyl)piperazine-1-ethanesulfonic acid (HEPES), 1.25 CaCl_2_, and 1 MgCl_2_ (Merck, Kenilworth, NJ, USA), buffered to pH = 7.4 with NaOH and has an osmolarity of 300 mOsm. AITC and A-967079 were obtained from Sigma-Aldrich (St. Louis, MO, USA). The abovementioned salts and HEPES were obtained from Carl Roth (Karlsruhe, Germany), Sigma-Aldrich (St. Louis, MO, USA) or Merck (Darmstadt, Germany), NaOH from Thermo Fisher scientific (Waltham, MA, USA).

### 4.7. Statistical Analysis

The statistical analysis of acquired experimental data was carried out using the GraphPad Prism v.9.1.0 software (GraphPad Software Inc., San Diego, CA, USA) and IBM SPSS statistics 24–26 (Armonk, New York, NY, USA). Statistical designs used a one-way ANOVA or ANCOVA with baselines as covariate. Comparison of cuprizone vs. control was prespecified, three further tests comparing additional treatments against cuprizone were corrected for inflation of the alpha error by Bonferroni-Holm correction for four tests. Baseline measurements as covariates were used to calculate the mean and 95% confidence interval estimates. *p* < 0.05 was considered significant. Percentage variations of experimental data between groups were calculated using Formula (1):

(1) $$\Delta \%=\frac{\mathrm{Mx}-\mathrm{My}}{\mathrm{My}}\times 100$$

where, Mx is the mean value for CPZ when compared vs. CTL, or CPZ + VEN, CPZ + RSP and CPZ + FEB groups when compared vs. CPZ; My is the mean value foreither CTL or CPZ.

## 5. Conclusions

Daily administration of venlafaxine, risperidone and febuxostat for five weeks reduced the effects of cuprizone-induced demyelination in mice, but only risperidone yielded significant results after histological analysis. Further research is required to establish their potential use in demyelinating diseases. Notably, febuxostat acts as a TRPA1 agonist at high concentrations, but it is unclear whether it binds in a covalent or non-covalent manner. Small molecules with similar structural features could be further investigated in order to identify novel TRPA1 agonists.

## Data Availability

The data presented in this study are available on request from the corresponding author.

## References

[1] Huang W.-J., Chen W.-W., Zhang X. (2017). Multiple sclerosis: Pathology, diagnosis and treatments. Exp. Ther. Med..

[2] Koch-Henriksen N., Sørensen P.S. (2010). The changing demographic pattern of multiple sclerosis epidemiology. Lancet Neurol..

[3] Loma I., Heyman R. (2011). Multiple sclerosis: Pathogenesis and treatment. Curr. Neuropharmacol..

[4] Pérez-Cerdá F., Sánchez-Gómez M.V., Matute C. (2016). The link of inflammation and neurodegeneration in progressive multiple sclerosis. Mult. Scler. Demyelinating Disord..

[5] Barnett M.H., Prineas J.W. (2004). Relapsing and Remitting Multiple Sclerosis: Pathology of the Newly Forming Lesion. Ann. Neurol..

[6] Triantafyllou N.I. (2003). Treatment of multiple sclerosis. Arch. Hell. Med..

[7] Gajofatto A., Benedetti M.D. (2015). Treatment strategies for multiple sclerosis: When to start, when to change, when to stop?. World J. Clin. Cases.

[8] Giovannoni G. (2017). Cladribine to Treat Relapsing Forms of Multiple Sclerosis. Neurotherapeutics.

[9] Nathoo N., Mackie A. (2017). Treating depression in multiple sclerosis with antidepressants: A brief review of clinical trials and exploration of clinical symptoms to guide treatment decisions. Mult. Scler. Relat. Disord..

[10] Pöllmann W., Feneberg W. (2008). Current management of pain associated with multiple sclerosis. CNS Drugs.

[11] Beiske G.A.G., Holmøy T., Beiske A.G., Johannessen S.I., Johannessen Landmark C. (2015). Antiepileptic and Antidepressive Polypharmacy in Patients with Multiple Sclerosis. Mult. Scler. Int..

[12] Dargahi N., Katsara M., Tselios T., Androutsou M.E., De Courten M., Matsoukas J., Apostolopoulos V. (2017). Multiple sclerosis: Immunopathology and treatment update. Brain Sci..

[13] Constantinescu C.S., Farooqi N., O’Brien K., Gran B. (2011). Experimental autoimmune encephalomyelitis (EAE) as a model for multiple sclerosis (MS). Br. J. Pharmacol..

[14] Zhan J., Mann T., Joost S., Behrangi N., Frank M., Kipp M. (2020). The Cuprizone Model: Dos and Do Nots. Cells.

[15] Zhen W., Liu A., Lu J., Zhang W., Tattersall D., Wang J. (2017). An Alternative Cuprizone-Induced Demyelination and Remyelination Mouse Model. ASN Neuro.

[16] Praet J., Guglielmetti C., Berneman Z., Van der Linden A., Ponsaerts P. (2014). Cellular and molecular neuropathology of the cuprizone mouse model: Clinical relevance for multiple sclerosis. Neurosci. Biobehav. Rev..

[17] Bölcskei K., Kriszta G., Sághy É., Payrits M., Sipos É., Vranesics A., Berente Z., Ábrahám H., Ács P., Komoly S. (2018). Behavioural alterations and morphological changes are attenuated by the lack of TRPA1 receptors in the cuprizone-induced demyelination model in mice. J. Neuroimmunol..

[18] Sághy É., Sipos É., Ács P., Bölcskei K., Pohóczky K., Kemény Á., Sándor Z., Szőke É., Sétáló G., Komoly S. (2016). TRPA1 deficiency is protective in cuprizone-induced demyelination—A new target against oligodendrocyte apoptosis. Glia.

[19] Moran M.M., Xu H., Clapham D.E. (2004). TRP ion channels in the nervous system. Curr. Opin. Neurobiol..

[20] Shigetomi E., Jackson-Weaver O., Huckstepp R.T., O’Dell T.J., Khakh B.S. (2013). TRPA1 channels are regulators of astrocyte basal calcium levels and long-term potentiation via constitutive d-serine release. J. Neurosci..

[21] Shigetomi E., Tong X., Kwan K.Y., Corey D.P., Khakh B.S. (2012). TRPA1 channels regulate astrocyte resting calcium and inhibitory synapse efficacy through GAT-3. Nat. Neurosci..

[22] Nilius B., Appendino G., Owsianik G. (2012). The transient receptor potential channel TRPA1: From gene to pathophysiology. Pflugers Arch. Eur. J. Physiol..

[23] Kheradpezhouh E., Choy J.M.C., Daria V.R., Arabzadeh E. (2017). TRPA1 expression and its functional activation in rodent cortex. Open Biol..

[24] Souza Monteiro de Araujo D., Nassini R., Geppetti P., De Logu F. (2020). TRPA1 as a therapeutic target for nociceptive pain. Expert Opin. Ther. Targets.

[25] Taylor-Clark T.E., Nassenstein C., McAlexander M.A., Undem B.J. (2009). TRPA1: A potential target for anti-tussive therapy. Pulm. Pharmacol. Ther..

[26] Andersson D.A., Gentry C., Moss S., Bevan S. (2008). Transient receptor potential A1 is a sensory receptor for multiple products of oxidative stress. J. Neurosci..

[27] Giorgi S., Nikolaeva-Koleva M., Alarcón-Alarcón D., Butrón L., González-Rodríguez S. (2019). Is TRPA1 burning down TRPV1 as druggable target for the treatment of chronic pain?. Int. J. Mol. Sci..

[28] Babes A., Ciotu C.I., Hoffmann T., Kichko T.I., Selescu T., Neacsu C., Sauer S.K., Reeh P.W., Fischer M.J.M. (2017). Photosensitization of TRPA1 and TRPV1 by 7-dehydrocholesterol: Implications for the Smith-Lemli-Opitz syndrome. Pain.

[29] Chen J., Hackos D.H. (2015). TRPA1 as a drug target—Promise and challenges. Naunyn. Schmiedebergs. Arch. Pharmacol..

[30] Mihai D.P., Trif C., Stancov G., Radulescu D., Nitulescu G.M. (2020). Artificial Intelligence Algorithms for Discovering New Active Compounds Targeting TRPA1 Pain Receptors. AI.

[31] Mihai D.P., Nitulescu G.M., Ion G.N.D., Ciotu C.I., Chirita C., Negres S. (2019). Computational drug repurposing algorithm targeting TRPA1 calcium channel as a potential therapeutic solution for multiple sclerosis. Pharmaceutics.

[32] Liebowitz M.R., Tourian K.A. (2010). Efficacy, Safety, and Tolerability of Desvenlafaxine 50 mg/d for the Treatment of Major Depressive Disorder:A Systematic Review of Clinical Trials. Prim. Care Companion J. Clin. Psychiatry.

[33] Morris M.T., Tarpada S.P. (2017). Long-Acting Injectable Paliperidone Palmitate: A Review of Efficacy and Safety. Psychopharmacol. Bull..

[34] Smith D., Dempster C., Glanville J., Freemantle N., Anderson I. (2002). Efficacy and tolerability of venlafaxine compared with selective serotonin reuptake inhibitors and other antidepressants: A meta-analysis. Br. J. Psychiatry.

[35] Li X., Zhu L., Su Y., Fang S. (2017). Short-term efficacy and tolerability of venlafaxine extended release in adults with generalized anxiety disorder without depression: A meta-analysis. PLoS ONE.

[36] Aiyer R., Barkin R.L., Bhatia A. (2017). Treatment of neuropathic pain with venlafaxine: A systematic review. Pain Med..

[37] Chen C.Y., Yeh Y.W., Kuo S.C., Liang C.S., Ho P.S., Huang C.C., Yen C.H., Shyu J.F., Lu R.B., Huang S.Y. (2018). Differences in immunomodulatory properties between venlafaxine and paroxetine in patients with major depressive disorder. Psychoneuroendocrinology.

[38] Li L., Zhang C. (2021). Venlafaxine Attenuated the Cognitive and Memory Deficit in Mice Exposed to Isoflurane Alone. Front. Neurol..

[39] Wang J., Qiao J., Zhang Y., Wang H., Zhu S., Zhang H., Hartle K., Guo H., Guo W., He J. (2014). Desvenlafaxine prevents white matter injury and improves the decreased phosphorylation of the rate-limiting enzyme of cholesterol synthesis in a chronic mouse model of depression. J. Neurochem..

[40] Chopko T.C., Lindsley C.W. (2018). Classics in Chemical Neuroscience: Risperidone. ACS Chem. Neurosci..

[41] MacDowell K.S., García-Bueno B., Madrigal J.L.M., Parellada M., Arango C., Micó J.A., Leza J.C. (2013). Risperidone normalizes increased inflammatory parameters and restores anti-inflammatory pathways in a model of neuroinflammation. Int. J. Neuropsychopharmacol..

[42] Kato T., Monji A., Hashioka S., Kanba S. (2007). Risperidone significantly inhibits interferon-γ-induced microglial activation in vitro. Schizophr. Res..

[43] Robinson P.C., Dalbeth N. (2018). Febuxostat for the treatment of hyperuricaemia in gout. Expert Opin. Pharmacother..

[44] Yan W., Zhang Y., Hu L., Li Q., Zhou H. (2021). Febuxostat Inhibits MPP+-Induced Inflammatory Response Through Inhibiting the JNK/NF-κB Pathway in Astrocytes. Neurotox. Res..

[45] Sangkuhl K., Stingl J.C., Turpeinen M., Altman R.B., Klein T.E. (2014). PharmGKB summary: Venlafaxine pathway. Pharmacogenet. Genom..

[46] Fang J., Bourin M., Baker G.B. (1999). Metabolism of risperidone to 9-hydroxyrisperidone by human cytochromes P450 2D6 and 3A4. Naunyn. Schmiedebergs. Arch. Pharmacol..

[47] Torkildsen Ø., Brunborg L.A., Myhr K.M., Bø L. (2008). The cuprizone model for demyelination. Acta Neurol. Scand..

[48] Liñares D., Taconis M., Maña P., Correcha M., Fordham S., Staykova M., Willenborg D.O. (2006). Neuronal nitric oxide synthase plays a key role in CNS demyelination. J. Neurosci..

[49] Sen M.K., Almuslehi M.S.M., Coorssen J.R., Mahns D.A., Shortland P.J. (2020). Behavioural and histological changes in cuprizone-fed mice. Brain. Behav. Immun..

[50] Liebetanz D., Merkler D. (2006). Effects of commissural de-and remyelination on motor skill behaviour in the cuprizone mouse model of multiple sclerosis. Exp. Neurol..

[51] Vollmar P., Nessler S., Kalluri S.R., Hartung H.P., Hemmer B. (2009). The antidepressant venlafaxine ameliorates murine experimental autoimmune encephalomyelitis by suppression of pro-inflammatory cytokines. Int. J. Neuropsychopharmacol..

[52] Vollmar P., Haghikia A., Dermietzel R., Faustmann P.M. (2008). Venlafaxine exhibits an anti-inflammatory effect in an inflammatory co-culture model. Int. J. Neuropsychopharmacol..

[53] Shen P., Hu Q., Dong M., Bai S., Liang Z., Chen Z., Li P., Hu Z., Zhong X., Zhu D. (2017). Venlafaxine exerts antidepressant effects possibly by activating MAPK-ERK1/2 and P13K-AKT pathways in the hippocampus. Behav. Brain Res..

[54] Cikánková T., Fišar Z., Bakhouche Y., Ľupták M., Hroudová J. (2019). In vitro effects of antipsychotics on mitochondrial respiration. Naunyn. Schmiedebergs. Arch. Pharmacol..

[55] Eftekhari A., Ahmadian E., Azarmi Y., Parvizpur A., Hamishehkar H., Eghbal M.A. (2016). In vitro/vivo studies towards mechanisms of risperidone-induced oxidative stress and the protective role of coenzyme Q10 and N-acetylcysteine. Toxicol. Mech. Methods.

[56] O’Sullivan D., Green L., Stone S., Zareie P., Kharkrang M., Fong D., Connor B., La Flamme A.C. (2014). Treatment with the antipsychotic agent, risperidone, reduces disease severity in experimental autoimmune encephalomyelitis. PLoS ONE.

[57] La Flamme A.C., Abernethy D., Sim D., Goode L., Lockhart M., Bourke D., Milner I., Garrill T.-M., Joshi P., Watson E. (2020). Safety and acceptability of clozapine and risperidone in progressive multiple sclerosis: A phase I, randomised, blinded, placebo-controlled trial. BMJ Neurol. Open.

[58] Miyata H., Takada T., Toyoda Y., Matsuo H., Ichida K., Suzuki H. (2016). Identification of febuxostat as a new strong ABCG2 inhibitor: Potential applications and risks in clinical situations. Front. Pharmacol..

[59] Honorat J.A., Kinoshita M., Okuno T., Takata K., Koda T., Tada S., Shirakura T., Fujimura H., Mochizuki H., Sakoda S. (2013). Xanthine Oxidase Mediates Axonal and Myelin Loss in a Murine Model of Multiple Sclerosis. PLoS ONE.

[60] Honorat J.A., Nakatsuji Y., Shimizu M., Kinoshita M., Sumi-Akamaru H., Sasaki T., Takata K., Koda T., Namba A., Yamashita K. (2017). Febuxostat ameliorates secondary progressive experimental autoimmune encephalomyelitis by restoring mitochondrial energy production in a GOT2-dependent manner. PLoS ONE.

[61] Aryanpour R., Pasbakhsh P., Zibara K., Namjoo Z., Beigi Boroujeni F., Shahbeigi S., Kashani I.R., Beyer C., Zendehdel A. (2017). Progesterone therapy induces an M1 to M2 switch in microglia phenotype and suppresses NLRP3 inflammasome in a cuprizone-induced demyelination mouse model. Int. Immunopharmacol..

[62] Zhang N., Zhang R., Loers G., Liu C., Jin L., Petridis A.K., Zheng X., Wang Z., Siebert H.C. (2020). Cuprizone-induced demyelination in mouse hippocampus is alleviated by ketogenic diet. J. Agric. Food Chem..

[63] Yoshikawa K., Palumbo S., Toscano C.D., Bosetti F. (2011). Inhibition of 5-lipoxygenase activity in mice during cuprizone-induced demyelination attenuates neuroinflammation, motor dysfunction and axonal damage. Prostaglandins Leukot. Essent. Fat. Acids.

[64] Yisireyili M., Hayashi M., Wu H., Uchida Y., Yamamoto K., Kikuchi R., Shoaib Hamrah M., Nakayama T., Wu Cheng X., Matsushita T. (2017). Xanthine oxidase inhibition by febuxostat attenuates stress-induced hyperuricemia, glucose dysmetabolism, and prothrombotic state in mice. Sci. Rep..

[65] Chiriță C., Ștefănescu E., Zbârcea C., Negreș S., Bratu M., Nuță D., Limban C., Chiriță I., Marineci C. (2019). Experimental pharmacological research regarding some new quinazolin-4-ones derivatives. J. Mind Med. Sci..

[66] Chiriță C., Ștefănescu E., Zbârcea C., Mireșan H., Negreș S., Nuță D., Limban C., Miulescu R., Marineci C. (2019). Experimental pharmacological research regarding the antidepressant effect of associating doxepin and selegiline in normal mice. J. Mind Med. Sci..

[67] Deacon R.M.J. (2013). Measuring motor coordination in mice. J. Vis. Exp..

[68] Deuis J.R., Dvorakova L.S., Vetter I. (2017). Methods used to evaluate pain behaviors in rodents. Front. Mol. Neurosci..

[69] Golden J.P., Hoshi M., Nassar M.A., Enomoto H., Wood J.N., Milbrandt J., Gereau IV R.W., Johnson E.M., Jain S. (2010). RET signaling is required for survival and normal function of nonpeptidergic nociceptors. J. Neurosci..

[70] Lippoldt E.K., Ongun S., Kusaka G.K., McKemy D.D. (2016). Inflammatory and neuropathic cold allodynia are selectively mediated by the neurotrophic factor receptor GFRα3. Proc. Natl. Acad. Sci. USA.

[71] Das Sarma J., Kenyon L.C., Hingley S.T., Shindler K.S. (2009). Mechanisms of primary axonal damage in a viral model of multiple sclerosis. J. Neurosci..

[72] Ungurianu A., Șeremet O., Grădinaru D., Ionescu-Tîrgoviște C., Margină D., Dănciulescu Miulescu R. (2019). Spectrophotometric versus spectrofluorometric assessment in the study of the relationships between lipid peroxidation and metabolic dysregulation. Chem. Biol. Drug Des..

[73] Katyare S.S., Rajan R.R. (2005). Influence of thyroid hormone treatment on the respiratory activity of cerebral mitochondria from hypothyroid rats. A critical re-assessment. Exp. Neurol..

[74] Margina D., Gradinaru D., Manda G., Neagoe I., Ilie M. (2013). Membranar effects exerted in vitro by polyphenols—Quercetin, epigallocatechin gallate and curcumin—On HUVEC and Jurkat cells, relevant for diabetes mellitus. Food Chem. Toxicol..

[75] Margină D., Olaru O.T., Ilie M., Grădinaru D., Guțu C., Voicu S., Dinischiotu A., Spandidos D.A., Tsatsakis A.M. (2015). Assessment of the potential health benefits of certain total extracts from Vitis vinifera, Aesculus hyppocastanum and Curcuma longa. Exp. Ther. Med..

[76] Gradinaru D., Margina D., Borsa C., Ionescu C., Ilie M., Costache M., Dinischiotu A., Prada G.I. (2017). Adiponectin: Possible link between metabolic stress and oxidative stress in the elderly. Aging Clin. Exp. Res..

[77] Zanfirescu A., Cristea A.N., Nitulescu G.M., Velescu B.S., Gradinaru D. (2018). Chronic monosodium glutamate administration induced hyperalgesia in mice. Nutrients.

[78] Miranda K.M., Espey M.G., Wink D.A. (2001). A rapid, simple spectrophotometric method for simultaneous detection of nitrate and nitrite. Nitric Oxide Biol. Chem..

[79] Nitulescu G., Mihai D.P., Nicorescu I.M., Olaru O.T., Ungurianu A., Zanfirescu A., Nitulescu G.M., Margina D. (2019). Discovery of natural naphthoquinones as sortase A inhibitors and potential anti-infective solutions against Staphylococcus aureus. Drug Dev. Res..

[80] Lowry O.H., Rosenbrough N.J., Farr A.L., Randall R.J. (1951). Protein measurement with the Folin phenol reagent. J. Biol. Chem..

[81] Thomas P., Smart T.G. (2005). HEK293 cell line: A vehicle for the expression of recombinant proteins. J. Pharmacol. Toxicol. Methods.

[82] McNamara C.R., Mandel-Brehm J., Bautista D.M., Siemens J., Deranian K.L., Zhao M., Hayward N.J., Chong J.A., Julius D., Moran M.M. (2007). TRPA1 mediates formalin-induced pain. Proc. Natl. Acad. Sci. USA.

[83] Hoebart C., Rojas-Galvan N.S., Ciotu C.I., Aykac I., Reissig L.F., Weninger W.J., Kiss A., Podesser B.K., Fischer M.J.M., Heber S. (2021). No functional TRPA1 in cardiomyocytes. Acta Physiol..

